# Supplementary material for: Causal Relationship between Aging and Anorexia Nervosa: A White-Matter-Microstructure-Mediated Mendelian Randomization Analysis
Source: Biomedicines. 2024 Aug 16;12(8):1874. doi: 10.3390/biomedicines12081874 (PMC11351342; doi:10.3390/biomedicines12081874)
Supplement: Supplementary file 1 [file biomedicines-12-01874-s001.zip › Supplementary Figure/Figure S4/Singe SNP plot of MR analysis for TL to ubm-b-2096.pdf]

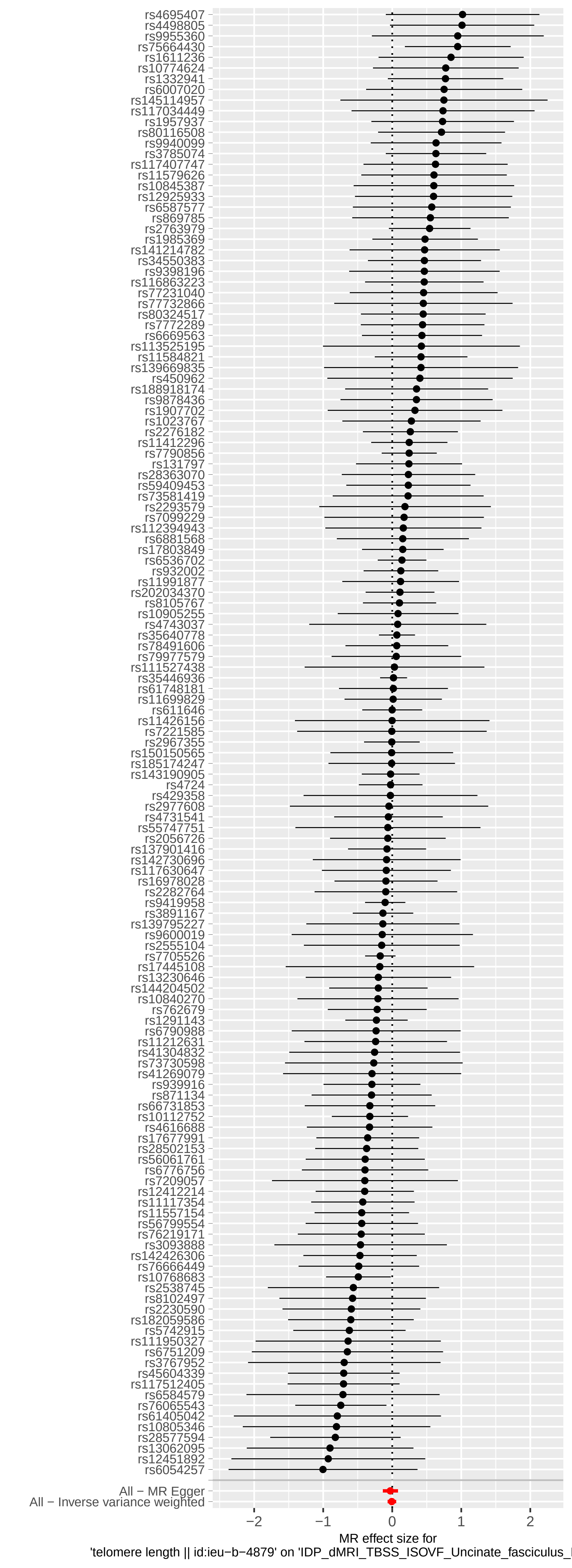

rs4695407
rs4498805
rs9955360
rs75664430
rs1611236
rs10774624
rs1332941
rs6007020
rs145114957
rs117034449
rs1957937
rs80116508
rs9940099
rs3785074
rs117407747
rs11579626
rs10845387
rs12925933
rs6587577
rs869785
rs2763979
rs1985369
rs141214782
rs34550383
rs9398196
rs116863223
rs77231040
rs77732866
rs80324517
rs7772289
rs6669563
rs113525195
rs11584821
rs139669835
rs450962
rs188918174
rs9878436
rs1907702
rs1023767
rs2276182
rs11412296
rs7790856
rs131797
rs28363070
rs59409453
rs73581419
rs2293579
rs7099229
rs112394943
rs6881568
rs17803849
rs6536702
rs932002
rs11991877
rs202034370
rs8105767
rs10905255
rs4743037
rs35640778
rs78491606
rs79977579
rs111527438
rs35446936
rs61748181
rs11699829
rs611646
rs11426156
rs7221585
rs2967355
rs150150565
rs185174247
rs143190905
rs4724
rs429358
rs2977608
rs4731541
rs55747751
rs2056726
rs137901416
rs142730696
rs117630647
rs16978028
rs2282764
rs9419958
rs3891167
rs139795227
rs9600019
rs2555104
rs7705526
rs17445108
rs13230646
rs144204502
rs10840270
rs762679
rs1291143
rs6790988
rs11212631
rs41304832
rs73730598
rs41269079
rs939916
rs871134
rs66731853
rs10112752
rs4616688
rs17677991
rs28502153
rs56061761
rs6776756
rs7209057
rs12412214
rs11117354
rs11557154
rs56799554
rs76219171
rs3093888
rs142426306
rs76666449
rs10768683
rs2538745
rs8102497
rs2230590
rs182059586
rs5742915
rs111950327
rs6751209
rs3767952
rs45604339
rs117512405
rs6584579
rs76065543
rs61405042
rs10805346
rs28577594
rs13062095
rs12451892
rs6054257
All − MR Egger
All − Inverse variance weighted
−2
−1
0
1
2
MR effect size for
'telomere length || id:ieu−b−4879' on 'IDP_dMRI_TBSS_ISOVF_Uncinate_fasciculus_
